# Supplementary material for: Intranasal trimeric sherpabody inhibits SARS-CoV-2 including recent immunoevasive Omicron subvariants
Source: Nat Commun. 2023 Mar 24;14:1637. doi: 10.1038/s41467-023-37290-6 (PMC10037368; doi:10.1038/s41467-023-37290-6)
Supplement: Supplementary file 1 — Supplementary Information [file 41467_2023_37290_MOESM1_ESM.pdf]

Supplementary data

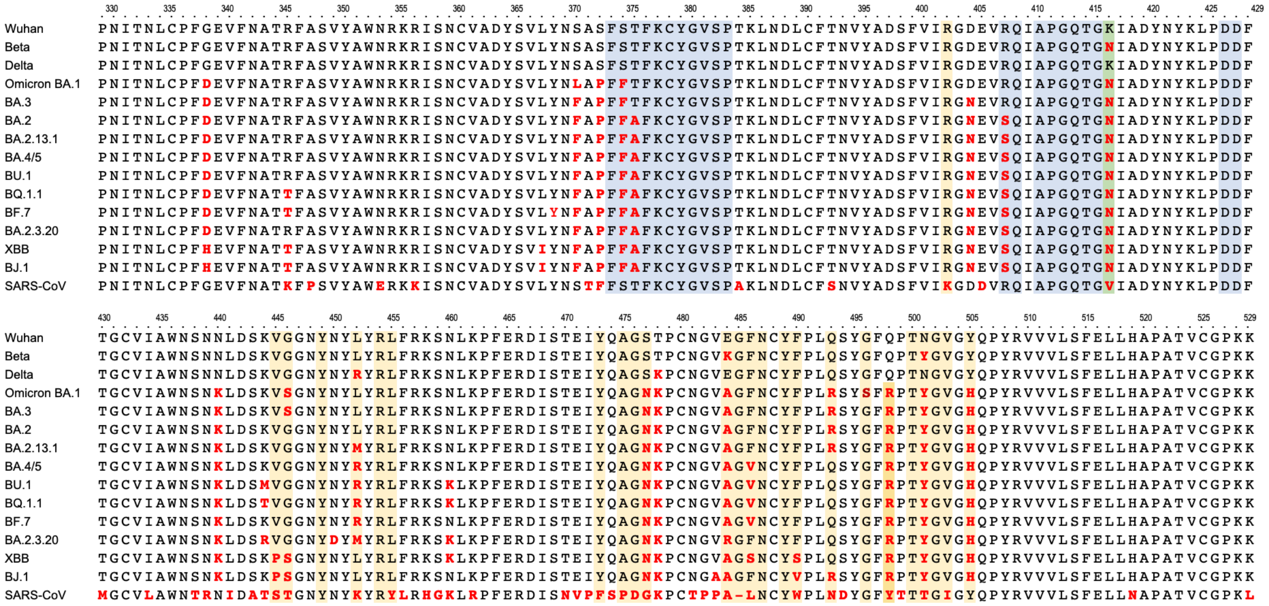

Supplementary Figure 1: Amino acid alignment of RBD region of SARS-CoV-2 and SARS-CoV-1 variants used in this study. Residues differing from the original SARS-CoV-2 Wuhan strain are indicated in red. Residues involved in the binding interfaces for TriSb92, ACE2, or both are highlighted in blue, yellow, or green, respectively. Source data are provided as a Source Data file.

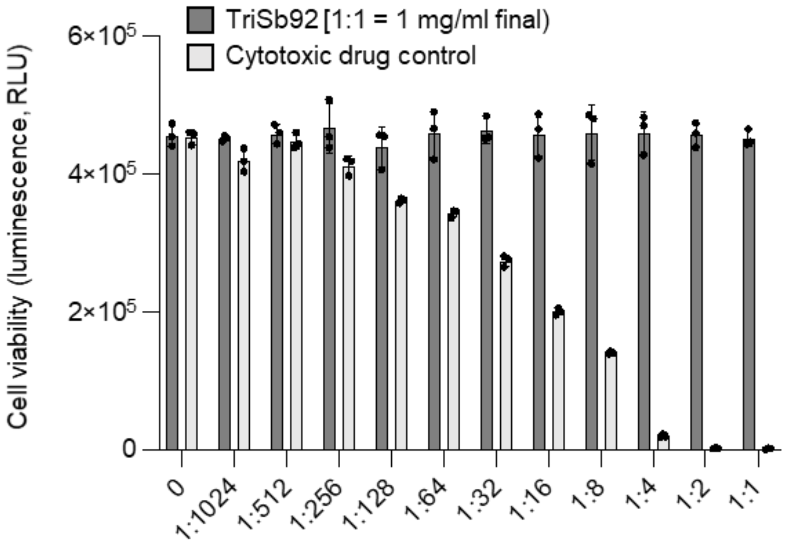

Supplementary Figure 2: TriSb92 shows no cytotoxicity. Viability of primary human nasal epithelial cells treated with increasing concentrations of TriSb92 for 24 h at 37°C. Cellular metabolic activity as a measure of cell viability was monitored using CellTiter-Glo 2.0 assay. The experiment was repeated independently three times with similar results. Average, standard deviations and the corresponding data points (n=3) of a representative assay performed in triplicates is shown. Source data are provided as a Source Data file.

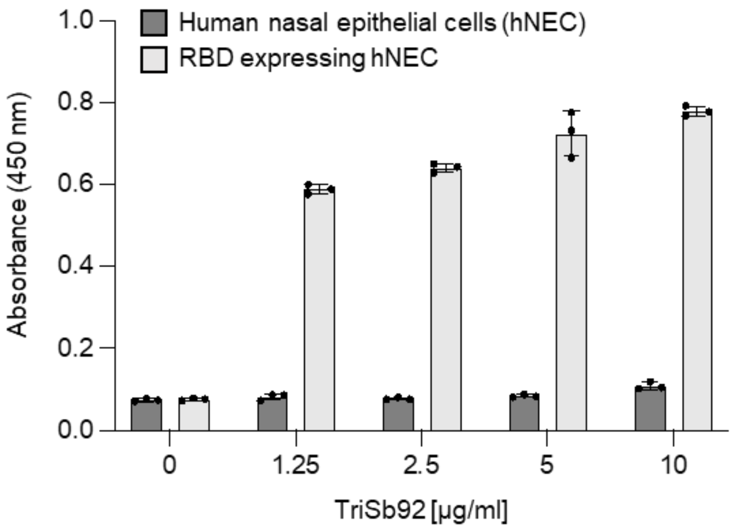

Supplementary Figure 3: Specificity of TriSb92 for RBD and lack of off-target binding to primary human nasal epithelial cell (hNECs) proteins. Binding of increasing concentrations of TriSb92 to hNEC lysates was tested in an ELISA-format. Lysate of the same cells transduced with an adenoviral vector expressing SARS-CoV-2 spike RBD were used as a positive control. The experiment was repeated independently three times with similar results. Average, standard deviations and the corresponding data points (n=3) of a representative assay performed in triplicates is shown. Source data are provided as a Source Data file.

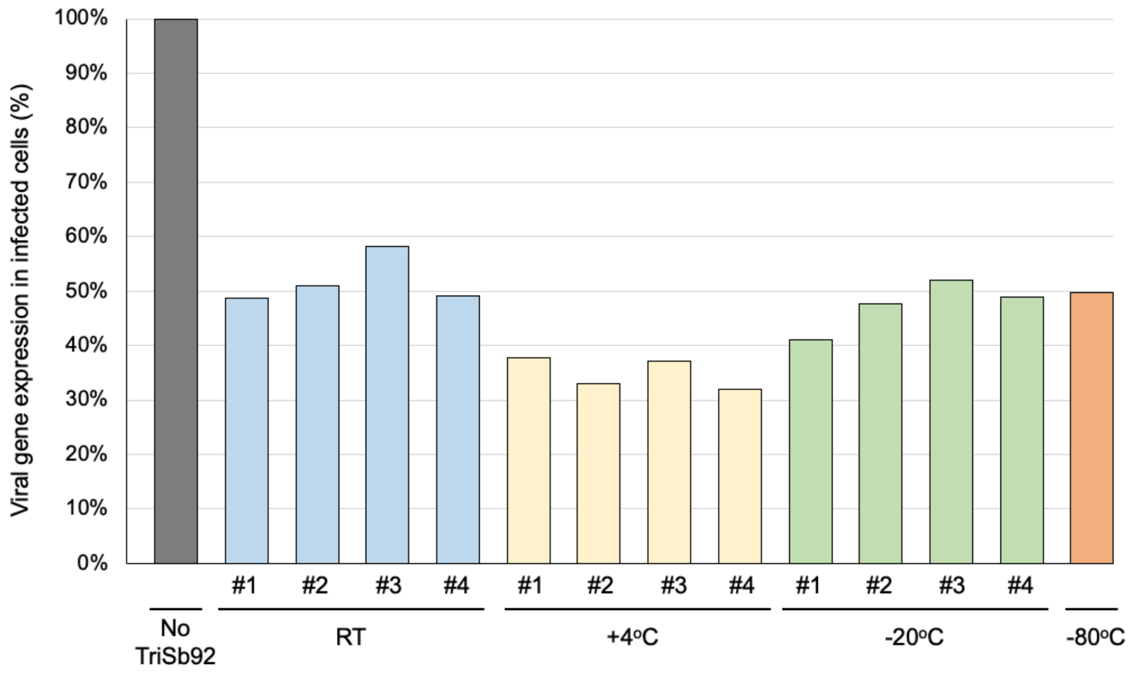

Supplementary Figure 4: Stability analysis of TriSb92 after long-term storage in different temperatures. Four independent aliquots of TriSb92 (1 mg/ml in PBS) stored for 15 months at the indicated temperatures were diluted to the IC_{50} concentration established for Wuhan spike-decorated pseudoviruses (see Fig. 2) and compared for their neutralization activity with TriSb92 stored at -80ºC. The dilutions were prepared based on the originally determined concentration without considering possible decrease in volume due to evaporation of samples stored at room temperature (RT) or +4ºC. Several storage temperature experiments were independently launched and subjected to repeated measurements revealing no loss of TriSb92 activity. Shown is one analysis performed in duplicate for samples from four series of such experiments initiated at consecutive weeks approximately 15 months earlier. Original pseudovirus neutralization data are provided as Source Data files.

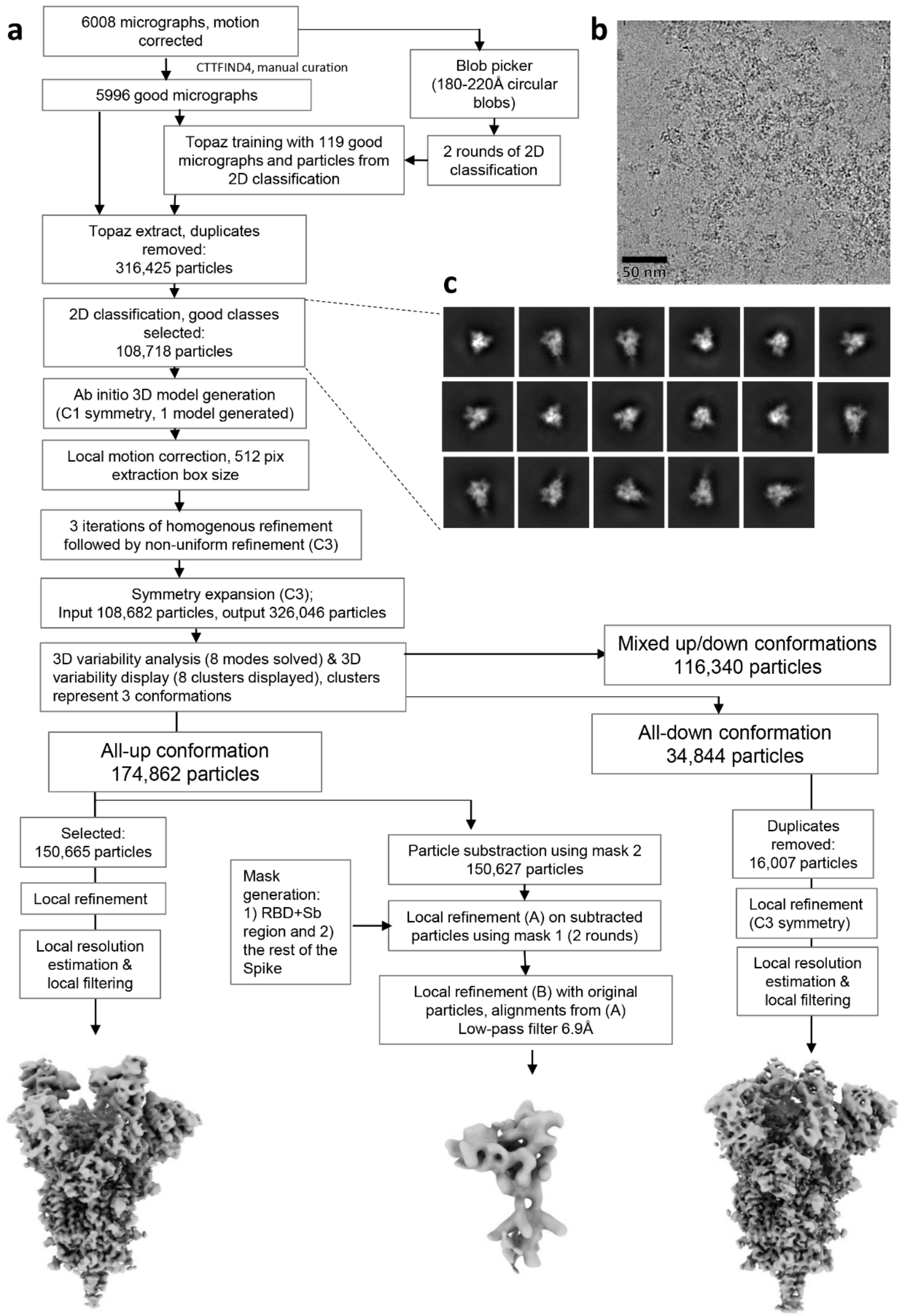

Supplementary Figure 5: CryoEM data processing. a Workflow for cryoEM data processing performed in cryoSPARC[1]. **b** A representative micrograph (of 6008 micrographs obtained) of TriSb92-treated S trimers. **c** 2D classes of the particles selected for ab initio model generation.

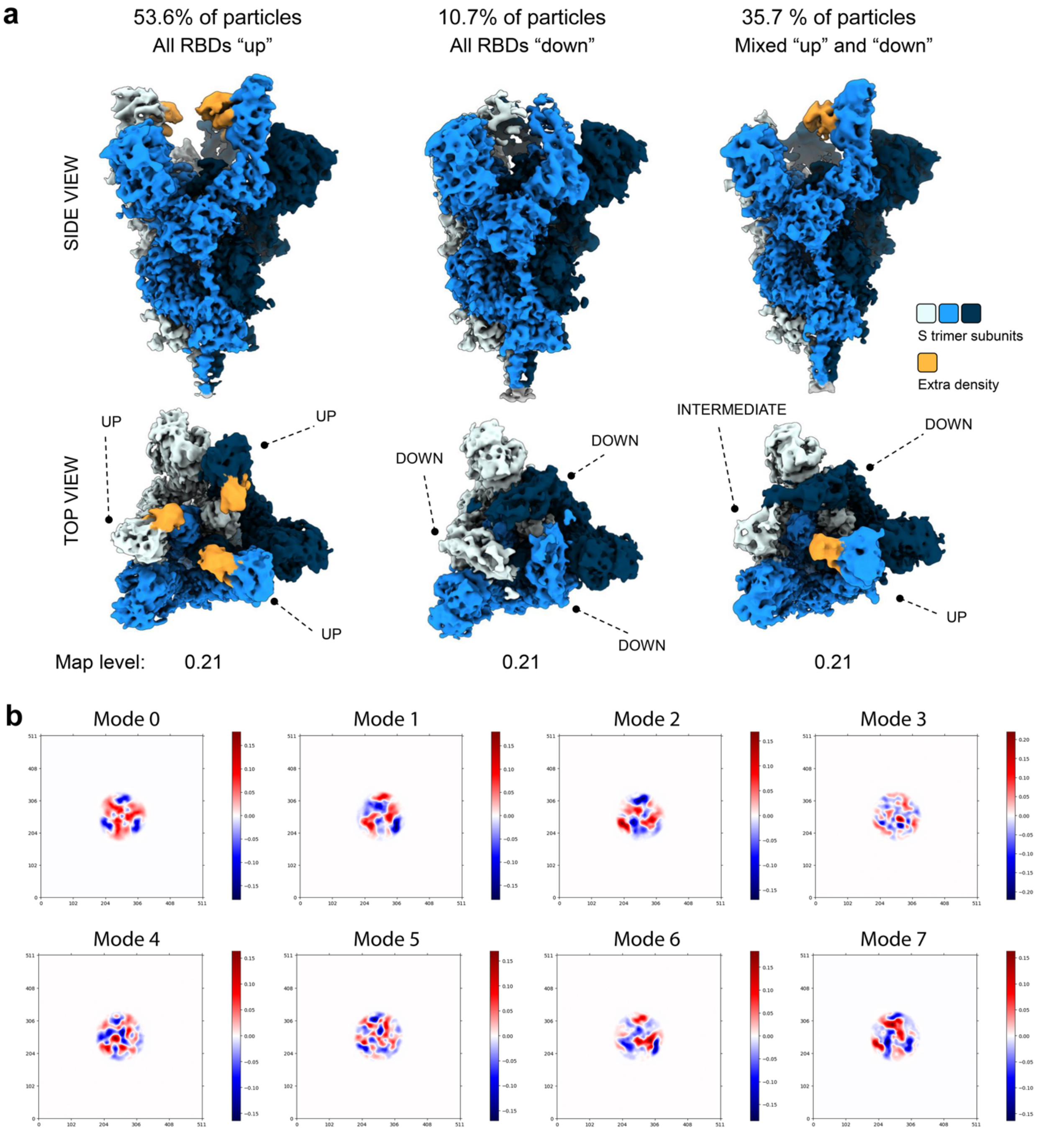

Supplementary Figure 6: CryoEM data shows conformationally distinct spike particle populations. **a** Particles were found to represent three main populations distinguished by RBD conformation: an allupmajority, and minority populations presenting an all down and mixed populations. Densities are colored in shades of blue for SARS-CoV-2 S-trimer subunits, and orange for the non-spike density which is observed adjacent to all up-conformation RBDs. **b** Variability component visualizations for the eight modes that were applied in 3D Variability Analysis[2] that identified conformational populations.

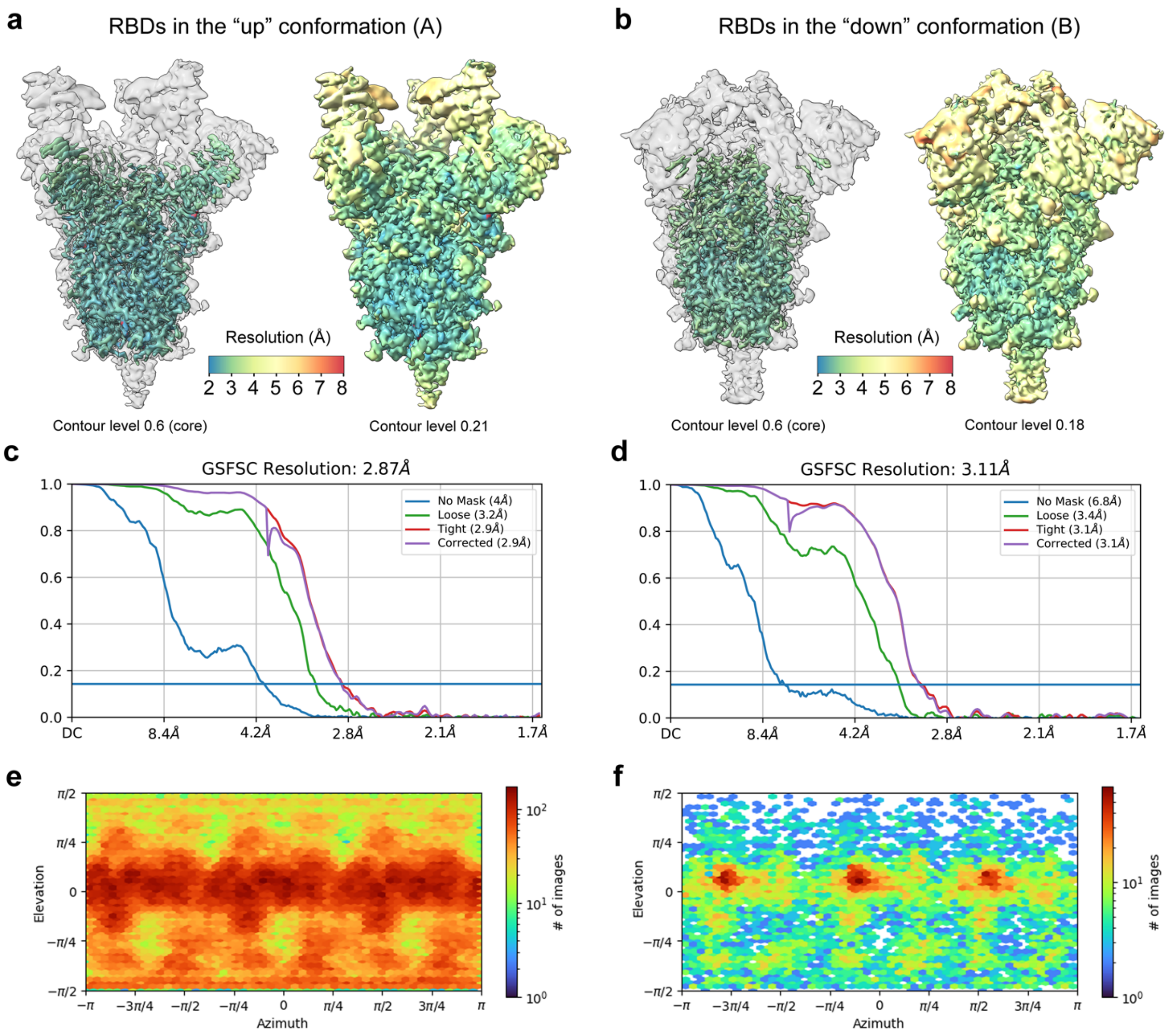

Supplementary Figure 7: CryoEM reconstructions derived from TriSb92-treated SARS-CoV-2 S trimers. Local resolution was estimated and maps were filtered to local resolution in cryoSPARC[1]. **a** Reconstruction A describes the S-trimer with all three RBDs in the up conformation, with an average estimated resolution of 2.9 Å, while **b** reconstruction B describes the S-trimer with all three RBDs in the down conformation and has average estimated resolution of 3.1 Å. The resolution is highest in the well-ordered S2 subunit, while the intrinsically flexible RBD regions display a more limited resolution. Localized refinement of the RBD regions produced a map at 6.9Å resolution (Fig S5 and S8). **c-d** Fourier shell correlation (FSC) plots, calculated between two half-maps as a function of spatial frequency, are shown for reconstructions A and B in panels **c** and **d**, respectively. **e-f** Viewing direction distribution plots for reconstructions A and B, respectively.

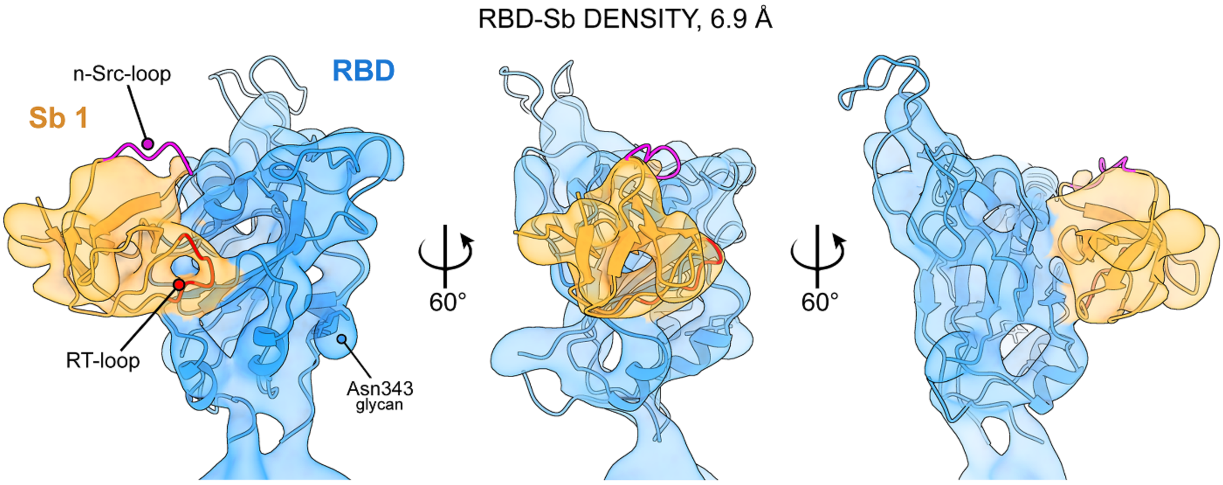

Supplementary Figure 8: Additional density observed in our cryoEM reconstruction matches with the predicted structure of Sb92. Cryo-EM reconstruction of the RBD-TriSb92 complex, with density assigned to the RBD shown in blue, and the density assigned to TriSb92 in orange. The RBD density region is shown with an RBD molecular model (blue, PDB 7A29; https://www.ncbi.nlm.nih.gov/Structure/pdb/7A29) and a model of Sb92 (generated with I-TASSER) fitted into the density. The RT- and n-Src loop regions of the SH3 fold that were modified for target-binding specificity (see Fig. 1) are indicated with red and magenta, respectively.

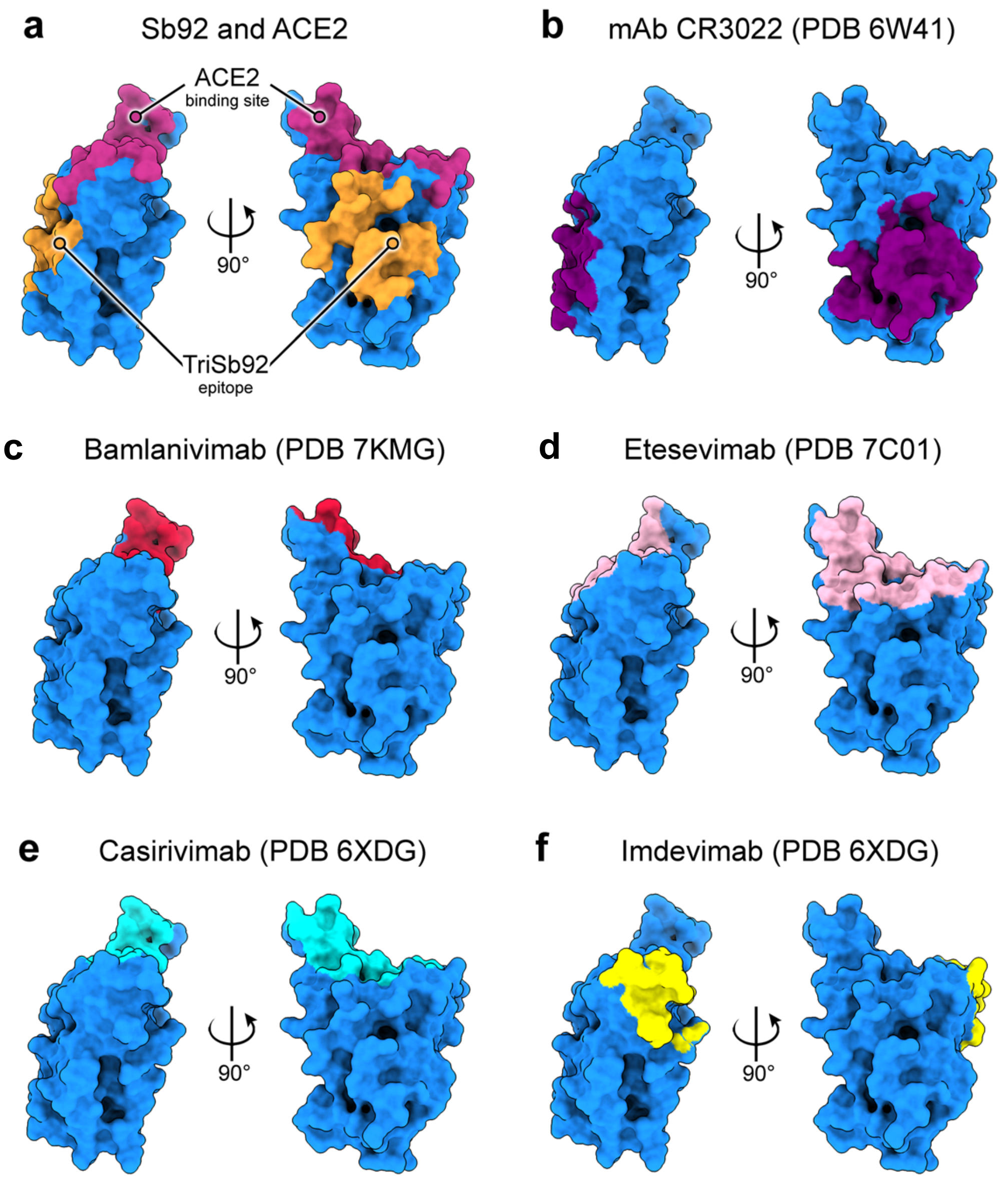

Supplementary Figure 9: Mapping Sb92 epitope in comparison to the epitopes of neutralizing mAbs. **a** Sb92 targets an epitope outside of the ACE2-binding region. **b** CR3022 (PDB 6W41; https://www.ncbi.nlm.nih.gov/Structure/pdb/6W41)[3], a SARS-CoV-1-neutralizing mAb capable of binding the SARS-CoV-2 RBD, utilizes a conserved and cryptic epitope that overlaps with the Sb92 epitope. **c-f** In contrast to Sb92, therapeutic monoclonal antibodies neutralizing SARS-CoV-2 typically target epitopes that overlap with the ACE2 binding site. These include Bamlanivimab (**c**, PDB 7KMG; https://www.ncbi.nlm.nih.gov/Structure/pdb/7KMG)[4], Etesevimab (**d**, PDB 7C01; https://www.ncbi.nlm.nih.gov/Structure/pdb/7C01)[5], Casirivimab (**e**, PDB 6XDG; https://www.ncbi.nlm.nih.gov/Structure/pdb/6XDG)[6] and Imdevimab (**f,** PDB 6XDG; https://www.ncbi.nlm.nih.gov/Structure/pdb/6XDG)[6].

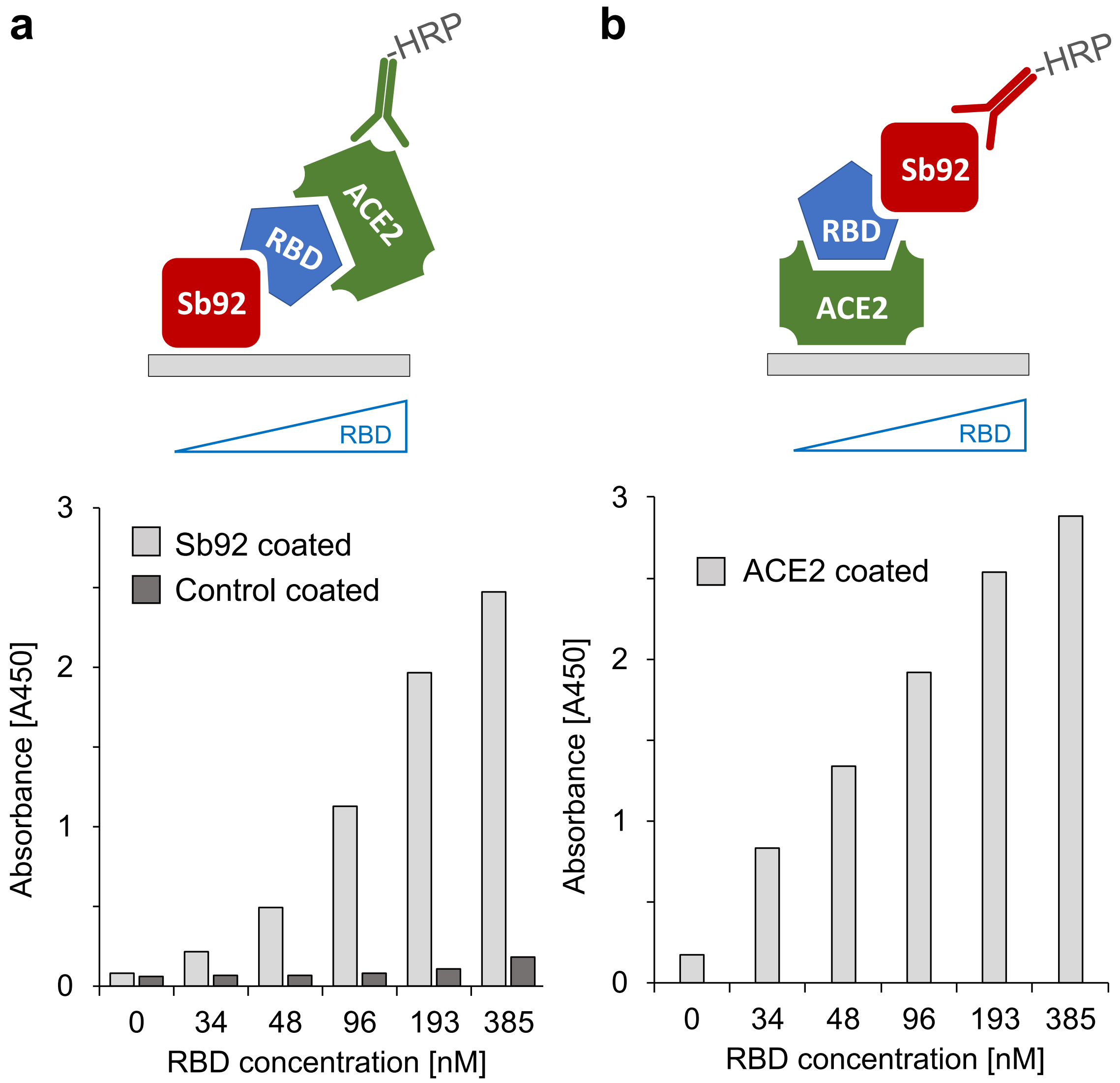

Supplementary Figure 10: Sandwich-ELISAs demonstrating simultaneous binding of Sb92 and ACE2 to RBD. ELISAs were performed in two ways: **a** using plastic-coated GST-Sb92 as the capturing reagent and ACE2-mFc as the detector reagent, or **b** vice versa. In panel **a**, a Her2-specific GST-Sb1206 was also tested as a negative control (Control coated). A constant amount of the capturing and detector reagents were used in each case, while increasing concentrations (0 – 385 nM) of RBD were added as a bridging reagent to test the formation of a trimeric Sb92-RBD-ACE2 complex, which was visualized using a HRP-conjugated antibody against the detector. The experiment was repeated independently three times with similar results. A representative assay is shown. Source data are provided as a Source Data file.

Supplementary Table 1. Cryo-EM data collection and processing statistics.

	S-trimer all RBDs up with TriSb92<br>EMD-16383 and PDB 8C1V	S-trimer all RBDs down<br>EMD-16388
**Data collection and processing**		
Magnification	165,000x	165,000x
Voltage (kV)	300	300
Electron exposure (e–/Å^2)	55	55
Defocus range (μm)	1.5–3.0	1.5–3.0
Pixel size (Å)	0.82	0.82
Symmetry imposed	C1	C3
Initial particle images (no.)	316,425	316,425
Final particle images (no.)	150,665 (C3 symmetry expanded)	16,007
Map resolution (Å)	2.87	3.11
FSC threshold	0.143	0.143

Supplementary Table 2. Summary of viral nucleoprotein immunohistochemistry findings in the mice included in Experiment 3 of Figure 3A.

Animal number	Treatment group	NP immunohistochemistry findings
B22-0089	Untreated	Lungs: All bronchioles positive, with few to all epithelial cells (EC) positive, viral antigen along luminal surface; next to some bronchioles or widespread patches/areas of alveoli with positive type I and II pneumocytes, and some free viral antigen in lumen. Trachea: individual positive EC. Bronchial lymph node (LN): Several positive macrophages/DC
B22-0090	Untreated	Lungs: Most bronchioles positive, with few to all EC positive, viral antigen along luminal surface; next to some bronchioles or numerous patches/areas of alveoli with positive type I and II pneumocytes, and some free viral antigen in lumen; Trachea: Individual positive EC and viral antigen along lumen
B22-0091	Untreated	Lungs: All bronchioles positive, with few to all EC positive, viral antigen along luminal surface; next to some bronchioles or widespread patches/areas of alveoli with positive type I and II pneumocytes, and some free viral antigen in lumen; Trachea: several individual positive EC, some viral antigen in lumen; Bronchial LN: several positive macrophages/DC
B22-0092	Untreated	Lungs: Many bronchioles with few to all EC positive, viral antigen along luminal surface; next to some bronchioles or widespread patches/areas of alveoli with positive type I and II pneumocytes, and some free viral antigen in lumen; Trachea: Several individual positive EC, some viral antigen in lumen; Bronchial LN: Several positive macrophages/DC
B22-0093	Untreated	Lungs: All bronchioles positive, with few to all EC positive, viral antigen along luminal surface; next to some bronchioles or widespread patches/areas of alveoli with positive type I and II pneumocytes and some free viral antigen in lumen; Trachea: Several individual positive EC, some viral antigen in lumen; Bronchial LN: Several positive macrophages/DC.
B22-0094	- 2h	Lungs: Negative; Trachea: Negative; Bronchial LN: Negative
B22-0095	- 2h	Lungs: Negative; Trachea: Negative; Bronchial LN: Negative
B22-0096	- 2h	Lungs: Negative; Trachea: Negative; Bronchial LN: Negative
B22-0097	- 2h	Lungs: Negative; Trachea: Negative; Bronchial LN: Negative
B22-0098	- 2h	Lungs: Negative; Trachea: Negative; Bronchial LN: Negative
B22-0099	+ 2h	Lungs: A few bronchioles with small to extensive patches of positive EC, viral antigen along luminal surface; a few small patches of alveoli with positive type I and II pneumocytes (mainly intact), also within consolidated areas; Bronchial LN: A few positive macrophages/DC

B22-0100	+ 2h	Lungs: A few individual and small patches of positive EC in bronchioles; a few small patches of alveoli with positive type I and II pneumocytes (intact)
B22-0101	+ 2h	Lungs: Several bronchioles with small to extensive patches of positive EC, viral antigen along luminal surface; several small to larger patches of alveoli with positive type I and II pneumocytes (mainly intact); Trachea: One patch of positive EC; Bronchial LN: Negative
B22-0102	+ 2h	Lungs: Several bronchioles with small to extensive patches of positive EC, some viral antigen along luminal surface; several small patches of alveoli with positive type I and II pneumocytes (mainly intact)
B22-0103	+ 2h	Lungs: Bronchioles with small to extensive patches of positive EC, viral antigen along luminal surface; several small patches of alveoli with positive type I and II pneumocytes (mainly intact), also within consolidated areas; Bronchial LN: A few positive macrophages/DC
B22-0104	+ 4h	Lungs: Several bronchioles with small to extensive patches of positive EC, viral antigen along luminal surface; several small patches of alveoli with positive type I and II pneumocytes (mainly intact); Trachea: Negative; Bronchial LN: Negative
B22-0105	+ 4h	Lungs: A few bronchioles with small to extensive patches of positive EC; a few small patches of alveoli with positive type I and II pneumocytes; Trachea: Negative; Bronchial LN: Negative
B22-0106	+ 4h	Lungs: A few bronchioles with small to extensive patches of positive EC and degenerate positive cells in lumen; a few small patches of alveoli with positive type I and II pneumocytes; Trachea: Negative
B22-0107	+ 4h	Lungs: Rare bronchioles with small to larger patches of positive EC; several small patches of alveoli with positive type I and II pneumocytes; Trachea: Negative
B22-0108	+ 4h	Lungs: Rare bronchioles with a few small patches of positive EC; a few small patches of alveoli with positive type I and II pneumocytes; Trachea: Negative
B22-0109	+ 8h	Lungs: Several bronchioles with a few individual to large patches of positive EC; several small patches of alveoli with positive type I and II pneumocytes
B22-0110	+ 8h	Lungs: A few bronchioles with a few individual to large patches of positive EC; several small patches of alveoli with positive type I and II pneumocytes; Bronchial LN: Negative
B22-0111	+ 8h	Lungs: several bronchioles with a few individual to large patches of positive EC; in one area a few small patches of alveoli with positive type I and II pneumocytes; Bronchial LN: Rare positive macrophages/DC
B22-0112	+ 8h	Lungs: several large bronchioles with many to all EC positive, viral antigen along luminal surface, a few bronchioles with a few individual or very small patches of positive EC; rare patches of alveoli with positive type I and II cells; Trachea: several individual positive EC, some viral antigen in lumen; Bronchial LN: A few positive macrophages/DC
B22-0113	+ 8h	Lungs: a few individual positive EC in bronchioles; several random small patches of alveoli with positive type I and II pneumocytes; Bronchial LN: Negative

Supplementary references

1. Punjani, A., Rubinstein, J.L., Fleet, D.J. & Brubaker, M.A. cryoSPARC: algorithms for rapid unsupervised cryo-EM structure determination. *Nat Methods* **14**, 290-296 (2017).

2. Punjani, A. & Fleet, D.J. 3D variability analysis: Resolving continuous flexibility and discrete heterogeneity from single particle cryo-EM. *J Struct Biol* **213**, 107702 (2021).

3. Yuan, M. et al. A highly conserved cryptic epitope in the receptor binding domains of SARS-CoV-2 and SARS-CoV. *Science* **368**, 630-633 (2020).

4. Jones, B.E. et al. The neutralizing antibody, LY-CoV555, protects against SARS-CoV-2 infection in nonhuman primates. *Sci Transl Med* **13** (2021).

5. Shi, R. et al. A human neutralizing antibody targets the receptor-binding site of SARS-CoV-2. *Nature* **584**, 120-124 (2020).

6. Hansen, J. et al. Studies in humanized mice and convalescent humans yield a SARS-CoV-2 antibody cocktail. *Science* **369**, 1010-1014 (2020).
