# Supplementary material for: Intranasal trimeric sherpabody inhibits SARS-CoV-2 including recent immunoevasive Omicron subvariants
Source: Nat Commun. 2023 Mar 24;14:1637. doi: 10.1038/s41467-023-37290-6 (PMC10037368; doi:10.1038/s41467-023-37290-6)
Supplement: Supplementary file 3 — Reporting Summary [file 41467_2023_37290_MOESM3_ESM.pdf]

nature portfolio

Corresponding author(s): Kalle Saksela
Last updated by author(s): Mar 9, 2023

Reporting Summary

Nature Portfolio wishes to improve the reproducibility of the work that we publish. This form provides structure for consistency and transparency in reporting. For further information on Nature Portfolio policies, see our Editorial Policies and the Editorial Policy Checklist.

Statistics

For all statistical analyses, confirm that the following items are present in the figure legend, table legend, main text, or Methods section.

n/a	Confirmed	
☐	☒	The exact sample size (*n*) for each experimental group/condition, given as a discrete number and unit of measurement
☐	☒	A statement on whether measurements were taken from distinct samples or whether the same sample was measured repeatedly
☒	☐	The statistical test(s) used AND whether they are one- or two-sided<br>*Only common tests should be described solely by name; describe more complex techniques in the Methods section.*
☒	☐	A description of all covariates tested
☒	☐	A description of any assumptions or corrections, such as tests of normality and adjustment for multiple comparisons
☐	☒	A full description of the statistical parameters including central tendency (e.g. means) or other basic estimates (e.g. regression coefficient) AND variation (e.g. standard deviation) or associated estimates of uncertainty (e.g. confidence intervals)
☒	☐	For null hypothesis testing, the test statistic (e.g. *F*, *t*, *r*) with confidence intervals, effect sizes, degrees of freedom and *P* value noted<br>*Give P values as exact values whenever suitable.*
☒	☐	For Bayesian analysis, information on the choice of priors and Markov chain Monte Carlo settings
☒	☐	For hierarchical and complex designs, identification of the appropriate level for tests and full reporting of outcomes
☒	☐	Estimates of effect sizes (e.g. Cohen's *d*, Pearson's *r*), indicating how they were calculated

Our web collection on statistics for biologists contains articles on many of the points above.

Software and code

Policy information about availability of computer code

Data collection	RT-qPCR was collected using AriaMx instrumentation (Agilent); Cryo-EM data were collected on a Titan Krios transmission electron microscope (Thermo Fisher Scientific) equipped with Gatan K2 direct electron detector using electron exposure of 55 e^{-}/Å^{2} per image at a nominal magnification of 165 000x, resulting in a pixel size of 0.82 Å. CryoEM data collection software was EPU v2.11.0.
Data analysis	Cryo-EM data were processed in cryoSPARC v4.1.1, using Topaz v0.2.3 for particle picking. A structural model for sherpabody Sb92 was predicted with the I-TASSER On-line server (corresponding downloadable package v5.1) and optimized with Phenix v1.19. Molecular modeling and structural analysis of SARS-CoV-2 spike densities were performed using UCSF Chimera v1.16, UCSF ChimeraX v 1.5, and Coot v0.9.6.2. The binding site of Sb92 on spike RBD was analysed with PDBePISA v1.52. Molecular graphics were generated using PyMOL (The PyMOL Molecular Graphics System, Version 2.5.0, Schrödinger, LLC), UCSF ChimeraX v 1.5, developed by the Resource for Biocomputing, Visualization, and Informatics at the University of California. Neutralization plots and IC50-values were processed with Microsoft Office Excel (2016).

For manuscripts utilizing custom algorithms or software that are central to the research but not yet described in published literature, software must be made available to editors and reviewers. We strongly encourage code deposition in a community repository (e.g. GitHub). See the Nature Portfolio guidelines for submitting code & software for further information.

Data

Policy information about availability of data

All manuscripts must include a data availability statement. This statement should provide the following information, where applicable:

- Accession codes, unique identifiers, or web links for publicly available datasets
- A description of any restrictions on data availability
- For clinical datasets or third party data, please ensure that the statement adheres to our policy

The cryo-EM density maps of i) SARS-CoV-2 spike with RBDs in the "up"-conformation and decorated with TriSb92 (accession code EMD-16383) and ii) SARS-CoV-2 spike with RBDs in the "down" conformation (EMD-16388) have been deposited in the EMDB at the EBI. Coordinates of the fitted SARS-CoV-2 spike in complex with Sb92 have deposited in the PDB (accession code PDB 8C1V). All data generated or analysed during this study are included in the main text and its supplementary information files. Other structures used in this study were obtained from the PDB with accession codes 1S1N (nephrocystin SH3 domain), 7KMS (SARS-CoV-2 S-trimer with all RBDs in the 'up' conformation bound to ACE2), 6ZP0 (SARS-CoV-2 S-trimer with all RBDs in the 'down' conformation), 7A29 (sybody-bound SARS-CoV-2 spike), and 6W41, 7KMG, 7C01, 6XDG (RBD-Fab complexes). PDB and EMDB depositions will be released upon publication of the manuscript (HPUB).

Human research participants

Policy information about studies involving human research participants and Sex and Gender in Research.

Reporting on sex and gender	This study did not involve human participants.
Population characteristics	This study did not involve human participants.
Recruitment	This study did not involve human participants.
Ethics oversight	This study did not involve human participants.

Note that full information on the approval of the study protocol must also be provided in the manuscript.

Field-specific reporting

Please select the one below that is the best fit for your research. If you are not sure, read the appropriate sections before making your selection.

☒ Life sciences ☐ Behavioural & social sciences ☐ Ecological, evolutionary & environmental sciences

For a reference copy of the document with all sections, see nature.com/documents/nr-reporting-summary-flat.pdf

Life sciences study design

All studies must disclose on these points even when the disclosure is negative.

Sample size	In this case the question of sample size relates mostly to the number of mice used in our experiments. According to the 3R principle of animal experimentation the number of animals used was as low as considered possible for obtaining unambiguous results (4 - 5 per treatment group). This was based on the expectation that the effects of the treatments tested would be categorical obvious without statistical analyses, which turned out to be true.<br><br>For experiments other than those involving mice, a maximum possible size of samples was included to allow scientific signifigance. The sample sizes are explained in the respective figure legends.
Data exclusions	No data were excluded from the analyses.
Replication	All data presented in the manuscript are reproducible. The excact replicate number is stated in the legends and methods. All attempts at replication of the results on the antiviral effects of our inhibitor in vivo (in mice) and in vitro (in cell culture) were successful, and no concerns regarding reproducibility could be identified.
Randomization	Not applicable or revevant. Our study design did not require and did not involve randomization.
Blinding	Mostly not applicable or relevant. Immunohistochemistry analyses of tissues from mice in different treatment groups were performed blinded to this group allocation.

Reporting for specific materials, systems and methods

We require information from authors about some types of materials, experimental systems and methods used in many studies. Here, indicate whether each material, system or method listed is relevant to your study. If you are not sure if a list item applies to your research, read the appropriate section before selecting a response.

Materials & experimental systems

n/a	Involved in the study
☐	☒ Antibodies
☐	☒ Eukaryotic cell lines
☒	☐ Palaeontology and archaeology
☐	☒ Animals and other organisms
☒	☐ Clinical data
☒	☐ Dual use research of concern

Methods

n/a	Involved in the study
☒	☐ ChIP-seq
☒	☐ Flow cytometry
☒	☐ MRI-based neuroimaging

Antibodies

Antibodies used	HRP-conjugated goat anti-Mouse IgG (Sigma Aldrich; Cat# 71045-3; Lot# SLCD0197); Goat Anti-E-tag-HRP conjugate (Bethyl; cat# A190132P; Lot# A190-232A); Mouse monoclonal anti-His(C-term)-HRP conjugate (Invitrogen; Cat# MA1-21315-HRP; Lot# 2312647); Mouse monoclonal anti-M13-HRP conjugate (GE Healthcare; Lot# 385982); Mouse monoclonal anti-GST-HRP conjugate (GE Healthcare/Cytiva; Cat# RPN1236; Lot# 17007382); Rabbit polyclonal anti-SARS-CoV nucleoprotein antibody (Rockland Immunochemicals; Cat# 200-401-A50; Lot# 200-402-A50); Goat Alexa Fluor 488-conjugated anti-rabbit polyclonal secondary antibody (Invitrogen; Cat# A-11008; Lot#2500542).
Validation	The commercial antibodies have been validated by the manufacturer.<br><br>Anti-E-tag antibody (GE Healthcare; cat# 27941201V; Lot# 355351)<br><br>Mouse monoclonal anti-His(C-term)-HRP conjugate (Invitrogen; Cat# MA1-21315-HRP; Lot# 2312647) https://www.fishersci.com/shop/products/anti-6x-his-epitope-tag-clone-his-h8-thermo-scientific-pierce-2/MA121315HRP<br><br>Mouse monoclonal anti-M13-HRP conjugate (GE Healthcare/Cytiva; Cat# RPN1236; Lot# 385982)<br>No longer commercially available.<br><br>Mouse monoclonal anti-GST-HRP conjugate (GE Healthcare; Lot# 17007382)<br>https://www.cytivalifesciences.com/en/us/shop/protein-analysis/blotting-and-detection/blotting-standards-and-reagents/anti-gst-hrp-conjugate-p-05747#overview<br><br>Rabbit polyclonal anti-SARS-CoV nucleoprotein antibody (Rockland Immunochemicals; Cat# 200-401-A50; Lot# 200-402-A50). https://www.rockland.com/categories/primary-antibodies/sars-nucleocapsid-protein-antibody-200-401-A50/

Eukaryotic cell lines

Policy information about cell lines and Sex and Gender in Research

Cell line source(s)	HEK293T (ATCC CRL-3216), HEK293T-ACE2 (this study), VeroE6-TMPRSS2-H10 (Rusanen et al. 2021), Expi293F™ (A14528; Thermo Fisher Scientific)
Authentication	All cell lines used had a well traceable origin but were authenticated in our laboratory by light microscopic morphological analysis only.
Mycoplasma contamination	All cells lines have been tested negative for mycoplasma contamination.
Commonly misidentified lines (See ICLAC register)	No commonly misidentified cells were used in the study.

Animals and other research organisms

Policy information about studies involving animals; ARRIVE guidelines recommended for reporting animal research, and Sex and Gender in Research

Laboratory animals	A total of 78 9 week old female Balb/c mice (from Envigo) were used in this study. The mice were kept 4-6 h in the light during the day time, and the rest of the day i.e. 18-20 h in the dark. The temperature and humidity in the BSL3 laboratory and cages was kept between 21-23 celsius and 25-35%, respectivley.
Wild animals	No wild animals was used
Reporting on sex	A total of 78 9 week old female Balb/c mice (from Envigo) were used in this study.

Field-collected samples	The study did not involve field-collected samples
Ethics oversight	Animal expreiments were approved by the Animal Experimental Board of Finland (license number ESAVI/28687/2020).

Note that full information on the approval of the study protocol must also be provided in the manuscript.
